# Glutathione as a powerful antioxidant in oxidative stress in the brain tissue of rats caused by the pathophysiological action of copper

**DOI:** 10.1192/j.eurpsy.2021.1906

**Published:** 2021-08-13

**Authors:** J. Jovanovic Mirkovic, Z. Jurinjak, G. Kocic, N. Medojevic, C. Alexopoulos

**Affiliations:** 1 Health Studies, The Academy of Applied Preschool Teaching and Health Studies, cuprija, Serbia; 2 Biochemistry, Faculty of medicine Nis, nis, Serbia; 3 Urology, Clinical Center Nis, nis, Serbia

**Keywords:** DNase, Copper, Brain of rats, glutathione

## Abstract

**Introduction:**

Due to increased human activity, the amount of copper in air, soil and water has increased. Copper, at minimum concentrations, is essential for the normal functioning of the organism (cellular respiration, hemoglobin formation, growth and reproduction). At higher concentrations, copper is deposited in the liver, brain tissue, and bone marrow.

**Objectives:**

To investigate the protective role of the supplement, glutathione (GSH), the S-donor ligand, in conditions of chronic copper intoxication via the parameters of oxidative stress, ie. Alkaline and acidic DNase values in brain tissue in albino rats of Wistar strain.

**Methods:**

The model system for testing the effects of copper exposure and the protective effect of GSH was a study on female albino rats of Wistar strain, stored in the vivarium of the Scientific Research Center for Biomedicine, Faculty of Medicine, Niš, Serbia. Endonucleases, alkaline and acidic DNase activities were determined spectrophotometrically from homogenates of brain tissue.

**Results:**

Copper is believed to be a likely cause of oxidative damage to the DNA molecule, as manifested by increased alkaline and acidic DNase activity. The results of this study show that GSH is a potent chelator that binds copper and enables its elimination from the body.

**Conclusions:**

In this experiment, the beneficial role of GSH supplements, which has an antioxidant character, in the prevention and reduction of the adverse effects of chronic copper intoxication was demonstrated. In this way, GSH acts as a powerful protector and antioxidant.

**Disclosure:**

No significant relationships.

